# The failed Latarjet: don't forget the Putti–Platt procedure: a case report

**DOI:** 10.1016/j.xrrt.2025.05.019

**Published:** 2025-06-10

**Authors:** Pascal Boileau, Manon Biegun, Gregorio Secci, Philipp Schippers, Mark Mouchantaf

**Affiliations:** a Department of Orthopaedic Surgery, ICR - Institut de Chirurgie Réparatrice, Locomoteur & Sports, Nice, France; b Department of Orthopaedic and Trauma Surgery, Centre Hospitalier de Haguenau, Haguenau, France

**Keywords:** Putti–Platt, Failed Latarjet, Reccurence of instability, Failed Bankart, Subscapularis elongation, Salvage procedure, Anterior shoulder dislocation, Glenohumeral instability

Surgical management of recurrence of anterior shoulder instability after a failed Latarjet procedure, in the absence of bony lesions, poses a significant challenge. We present the case of a 21-year-old young patient who underwent a Latarjet procedure to try to stabilize the shoulder after a failed Bankart repair. The patient presented with recurrence of anterior shoulder instability following the Latarjet procedure despite a well-positioned and fully healed coracoid bone block and no Hill-Sachs lesion. A third surgical intervention was performed to carry out an open capsular shift after the previous screw removal had failed to resolve the issue of recurrent anterior instability.

## Case presentation

### Clinical presentation and imaging findings

A 18-year-old young woman experienced a first traumatic anterior shoulder dislocation of the right shoulder in February 2017 during a gymnastics session; the anterior dislocation was reduced by her coach. In September 2017, she underwent an arthroscopic Bankart repair for shoulder stabilization but sustained a new anterior shoulder dislocation during volleyball 3 months later. Following the failure of the Bankart repair, she subsequently underwent an open Latarjet procedure. Unfortunately, this revision procedure also failed, leading to a painful recurrence of subluxations. In June 2018, the patient underwent a third surgical procedure to perform a capsular shift and remove the screws, but this revision surgery still did not resolve the issue of recurrent anterior instability. In 2021, she received a Botox injection in the pectoralis major muscle to address static anterior shoulder subluxation, but this intervention showed no improvement either.

**Figure 1:**
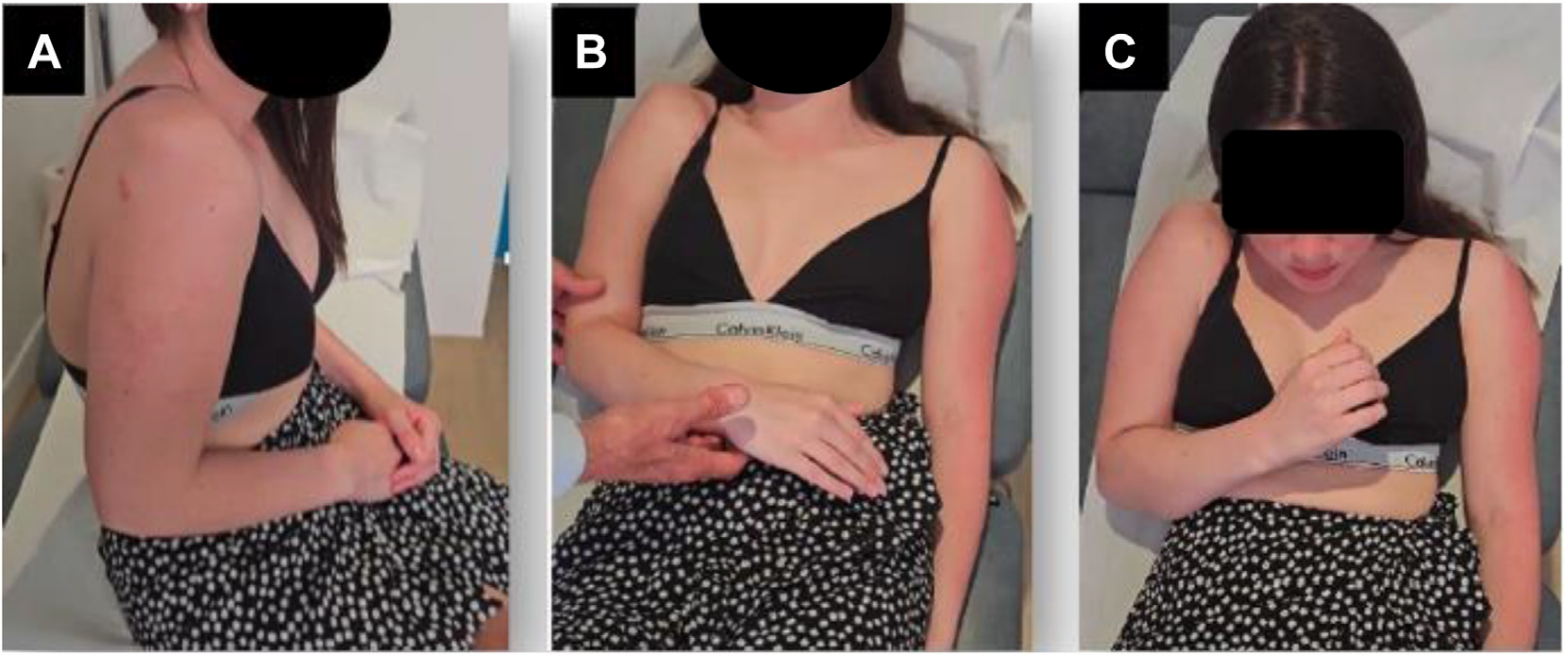
Clinical presentation: 21-year-old lady with an unstable shoulder after 3 previous failed surgeries: Failed arthroscopic Bankart, failed open Latarjet and failed capsular shift with screw removal (**A**) the permanent anterior subluxation of the right shoulder is visible; (**B**) the arm is kept in internal rotation because of painful recurrent subluxations, and (**C**) the patient uses only her elbow and wrist to reach her mouth.

By the time she presented to our clinic, the patient was 21 years old and had been living with a 3-year history of right anterior shoulder instability and pain following the 3 failed stabilization procedures. She described her life as miserable because of severe shoulder pain (visual analogue score = 10/10) and complete disability (shoulder subjective value = 0%). She was unable to perform daily activities, participate in sports, or move her shoulder, significantly impacting her social, professional, and personal life. On clinical examination, there was no deltoid atrophy, but the shoulder was permanently subluxed anteriorly. The patient kept her arm permanently in internal rotation and refused to move it due to severe pain, popping and subluxations (Fig. 1). To note, she had undergone an open Latarjet procedure on her contralateral (left) shoulder in 2016, yielding favorable clinical outcomes. Generalized hyperlaxity was also noted in this patient, with a Beighton score of 6 out of 9. It is worth mentioning that the examination of laxity in the right upper limb was limited due to disability and pain. She has never experienced any issues in any joint other than her shoulders.

Bilateral anteroposterior radiographs of the shoulders showed moderate osteoarthritis (Samilson grade 2) with anteroinferior subluxation of the humeral head on the right side and perfect positioning of the coracoid bone block on the left side. Computed tomography (CT) scan of the right shoulder confirmed static anterior subluxation of the humeral head despite a well-positioned and healed coracoid bone graft with no Hill–Sachs lesion and no atrophy or fatty infiltration of the subscapularis muscle. A psychiatric evaluation ruled out voluntary instability or a psychiatric disorder. A neurological consultation excluded the possibility of undiagnosed epilepsy, and an electromyogram ruled out axillary nerve palsy. Laboratory tests, including sedimentation rate and C-reactive protein, were normal, with no indication of low-grade infection.

In summary, this 21-year-old patient experienced recurrence of anterior instability despite having undergone a well-executed Latarjet procedure and 2 previous soft tissue interventions (1 arthroscopic Bankart repair and 1 open capsular shift). She presented with dynamic and static anterior subluxation of the humeral head, despite a perfectly positioned and healed coracoid bone block (flush with the glenoid surface and below the equator) and no evidence of humeral bone defects. After a comprehensive evaluation and considering the successful outcome of her prior Latarjet procedure on the left (contralateral) shoulder, we opted for surgical management. We hypothesized that the recurrence of instability could be due to subscapularis and anterior capsular elongation and insufficiency,^6^,^29^ and that a subscapularis and capsular shortening could provide a potential solution.^11^

### Management and surgical treatment

Under general anesthesia, with the patient in a semisitting position, the shoulder was found to be passively mobile with no stiffness. The load and shift test were positive (+3), and fluoroscopy confirmed the anterior direction of shoulder instability. Diagnostic arthroscopy confirmed some osteoarthritis and showed that the anterior labrum was absent with a capsule of poor quality and intact rotator cuff tendons. A modified Putti–Platt procedure was then performed through a deltopectoral approach. After exposing the tendon of the subscapularis muscle, the rotator interval was opened superiorly, and the anterior axillary vessels were ligated inferiorly.

**Figure 2:**
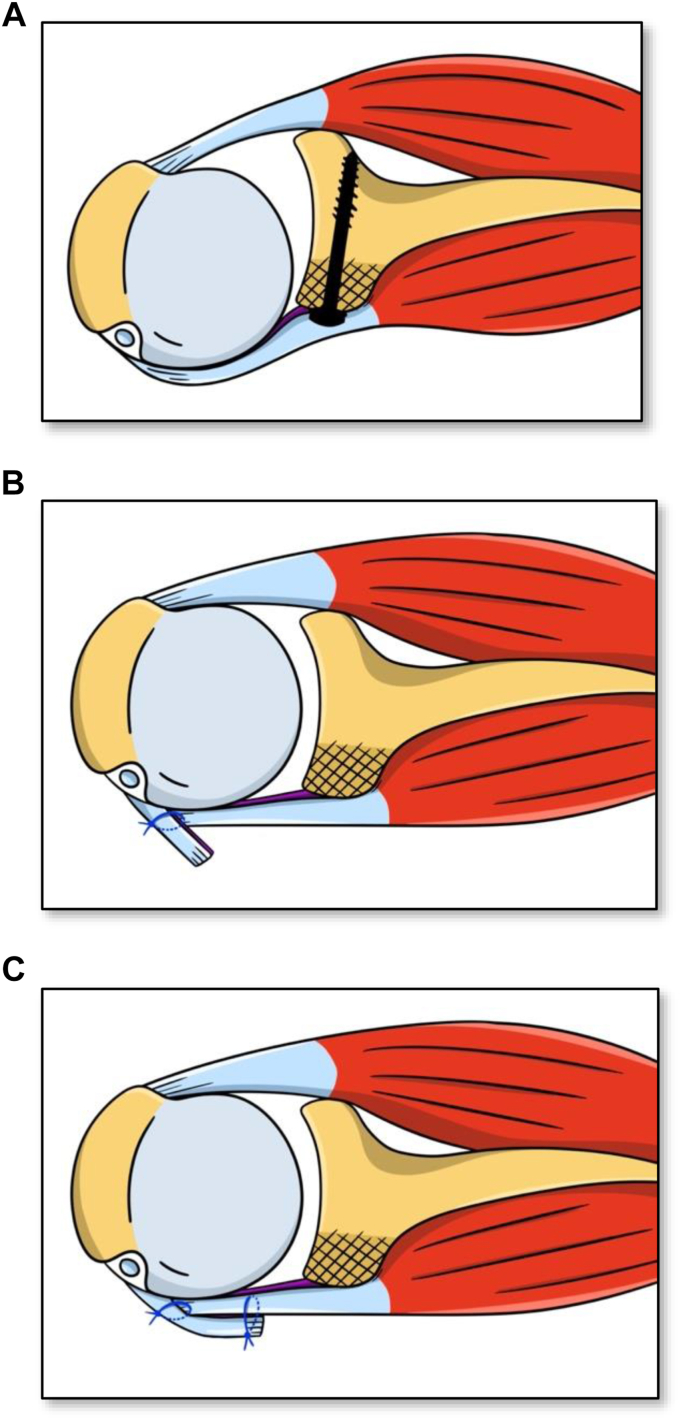
Drawing showing the modified Putti–Platt procedure. (**A**) Before revision surgery, the humeral head was permanently subluxed anteriorly because of the elongation of the anterior soft tissues; (**B**) To shorten the anterior soft tissues, the medial flap of capsule and tendon was passed under the lateral flap and sutured to the remaining tendon, medial to the bicipital groove; (**C**) the lateral flap (1.5-2 cm long) was then sutured over the subscapularis to perform a care coat.

**Figure 3:**
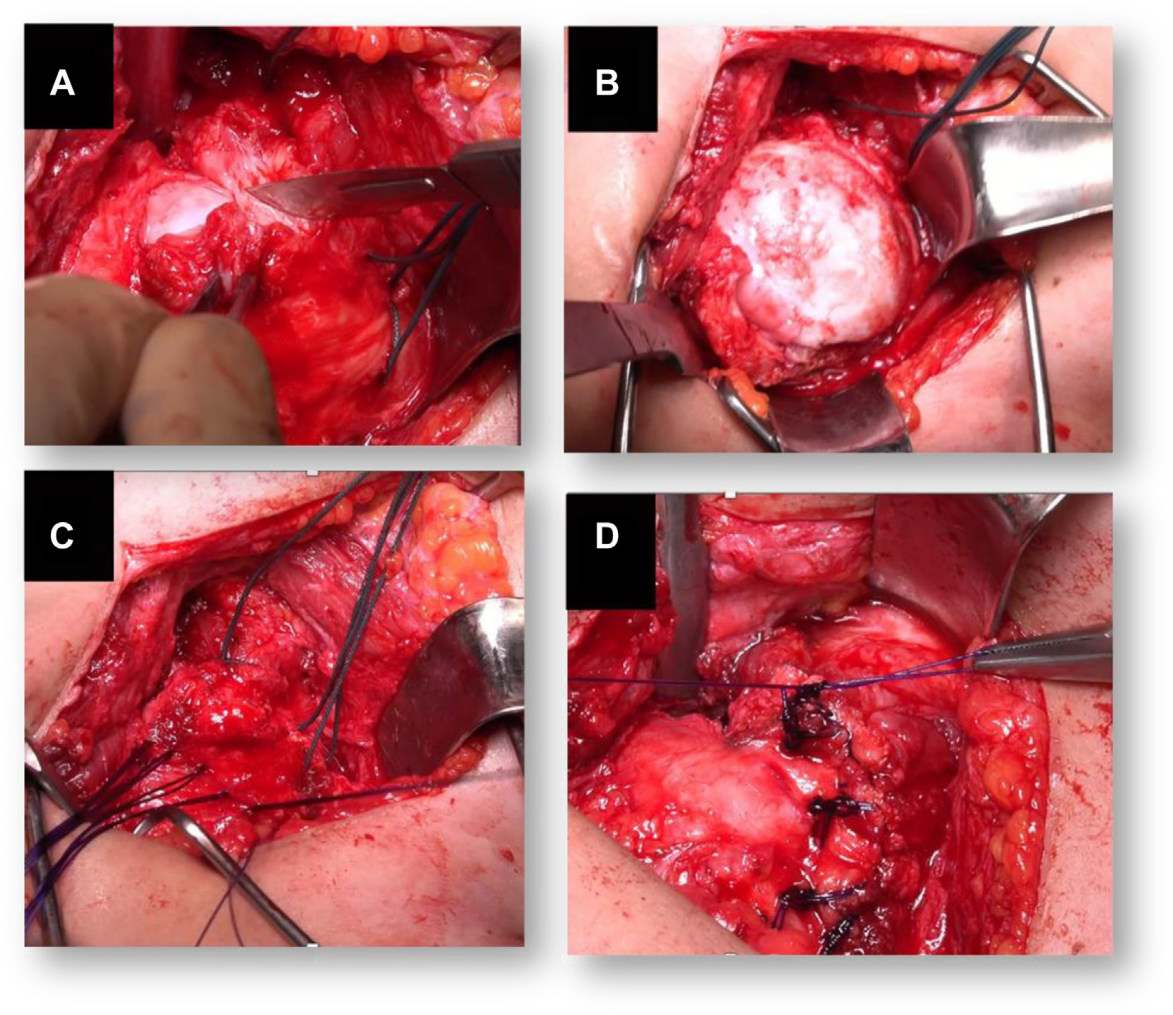
Intraoperative images showing the modified Putti–Platt procedure. (**A**) The subscapularis and capsule are incised together following the oblique line of the anatomical neck, leaving about 2 cm of tendon and capsule attached on the lesser tuberosity; (**B**) Exposition the humeral head which shows some osteoarthritis; (**C**) 4 PDS 2-0 sutures with Mason–Allen stiches were placed on the medial leaf and then passed as laterally as possible through the remaining lateral stump to make U-stitches (**D**). After tightening the 4 U-stitches to shorten the subscapularis and the anterior capsule, a continuous stitch of PDS 2-0 was used to secure the remaining lateral stump over the subscapularis muscle. *PDS*, polydioxanone suture.

In the original Putti–Platt procedure, the capsule is separated from the muscle and incised 1 cm more medial than the tendon and then reattached to the anterior labrum. In our procedure, the subscapularis tendon was not separated from the underlying capsule. The tendon and capsule were vertically transected together, 2 cm from their insertion on the lesser tuberosity; the medial flap was attached to the lesser tuberosity and the lateral flap was overlapping the medial flap (Fig. 2). The incision of tendon and capsule was oblique, following the anatomical neck of the humerus and extending inferiorly to the 6 o'clock position. A curette was used to roughen the bone of the lesser tuberosity and facilitate the healing of the medial flap to the bone. The subscapularis muscle and capsule were shortened by transferring the medial flap under the lateral flap and then covering the lateral flap over the medial flap (Fig. 3). Using Mason–Allen stitches, 4 polydioxanone suture-loop sutures were placed in the medial stump of the subscapularis and then passed as laterally as possible through the lateral stump in an inside-out manner to form U-stitches. The sutures were tightened, with the arm in neutral rotation. The remaining lateral stump was overlapped on the medial leaf and secured over the subscapularis muscle using a continuous polydioxanone suture suture. Finally, the rotator interval was closed laterally with 2 absorbable stiches.

Postoperatively, an abduction brace was put in place for a total of 6 weeks postoperatively to avoid adherence and stiffness. Self-rehabilitation and swimming pool therapy was recommended to avoid any potential complications.

## Clinical and radiological outcomes

**Figure 4:**
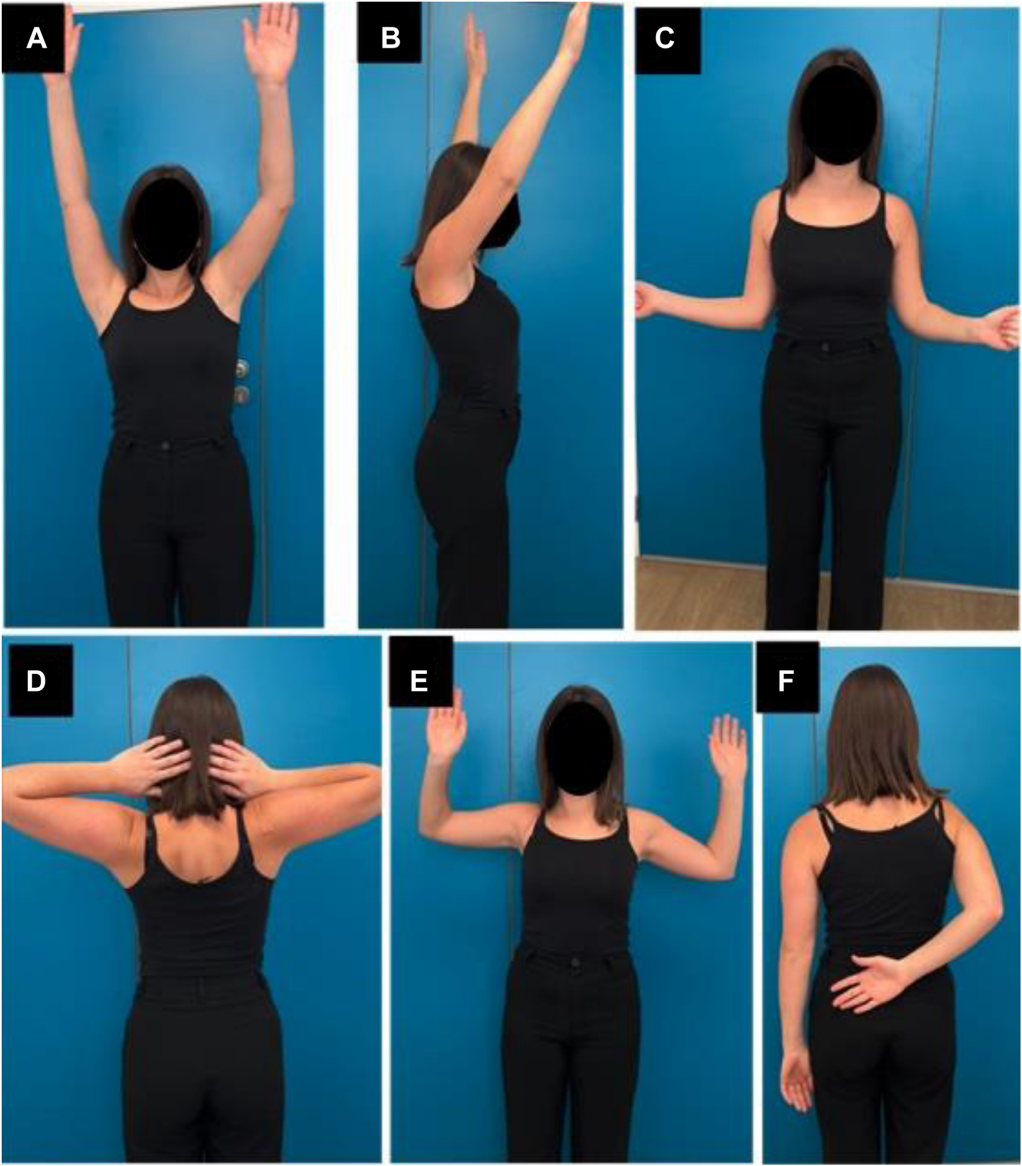
Postoperative range of motion images at 2 years of follow-up demonstrating almost normal active forward flexion (**A**) and (**B**), external rotation (**C**), hands behind the head with the elbows posteriorly (**D**) External rotation with arms at 90° of abduction (**E**), and internal rotation (**F**).

The patient had regular follow-up visits at 6 weeks, 3 months, 6 months, and 1 and 2 years postoperatively. At the latest follow-up of 2 years postoperatively, the patient was very satisfied with the procedure, with a shoulder subjective value of 90% and a visual analogue score of 2 of 10. She regained almost full range of motion in her shoulder (Fig. 4), with 160° in active forward elevation, 160° in abduction, 60° in external rotation, and internal rotation to L5. The Constant–Murley score improved from 0 to 70 points and the improvement of the Walch–Duplay score was from 0 to 70%.

**Figure 5:**
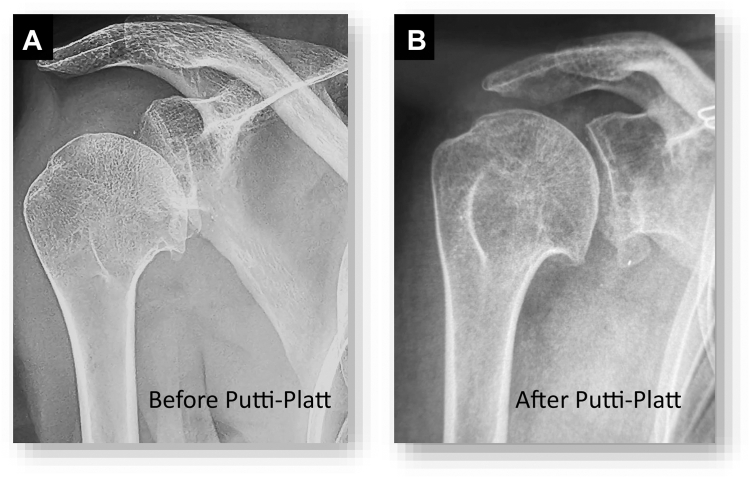
Preoperative and postoperative anteroposterior radiographs showing (**A**) before revision, there is anteroinferior subluxation of the humeral head despite a well-positioned and healed coracoid bone graft; (**B**) 2 years after revision with a modified Putti–Platt procedure, the humeral head is recentered, facing the glenoid and there is no increase of glenohumeral osteoarthritis.

**Figure 6:**
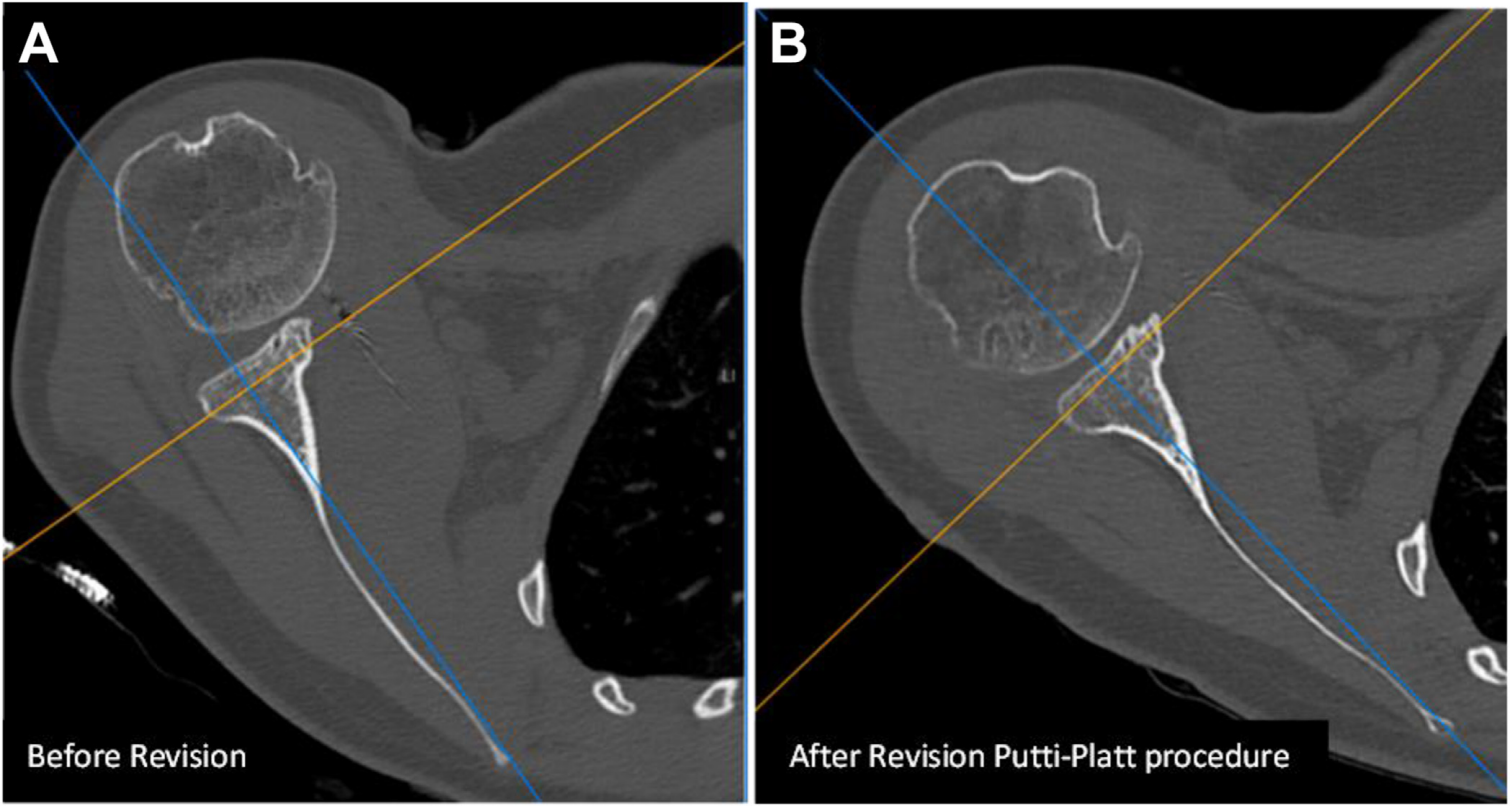
Comparison of preoperative and postoperative 2-dimensional Axial CT scan images. (**A**) Preoperatively, there is anterior subluxation of the humeral head despite good positioning and healing of coracoid bone block, absence of Hill-Sachs lesion and normal subscapularis muscle and (**B**) postoperatively, the humeral is recentered in front of the glenoid surface. Notice that the subscapularis muscle did not deteriorate, and osteoarthritis did not increase. *CT*, computed tomography.

**Figure 7:**
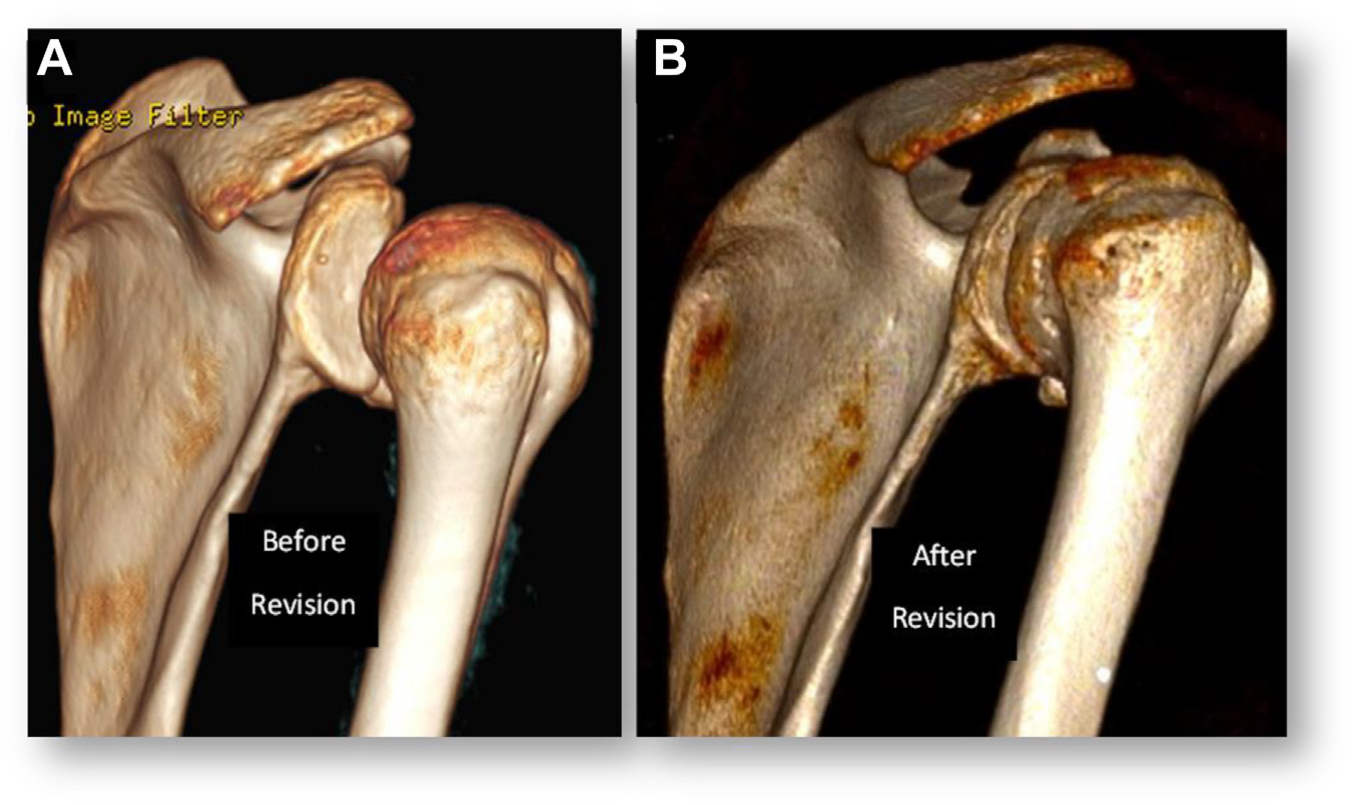
Preoperative and Postoperative 3-dimensional CT scan images showing (**A**) severe anterior subluxation of the humeral head before surgery and (**B**) well-centered humeral head after revision with the modified Putti–Platt procedure. *CT*, computed tomography.

As for the radiologic outcomes, anteroposterior (AP) showed a well-reduced glenohumeral joint with moderate signs of glenohumeral arthritis, Samilson grade 2 (Fig. 5). Interestingly, the CT scan at follow-up showed a well-centered humeral head according to the scapular axis, compared to the preoperative scan where the head was subluxed anteriorly (Figs. 6 and 7). The patient was encouraged to resume her usual activities, continue physical therapy and swimming to strengthen her shoulder.

## Discussion

The Latarjet procedure, which involves transferring the coracoid process to the glenoid, is often employed to assure shoulder stability in young athletes^7^,^23^,^30^ and in case of failure of the Bankart procedure.^1^, ^2^, ^3^^,^^20^ Recurrence of instability after Latarjet procedure is rare and most of the time related to technical issues with malposition, nonunion or lysis of the bone block or to the presence of a large, deep Hill–Sachs lesion.^5^^,^^9^^,^^14^ Recurrence of anterior instability after a Latarjet procedure, with correct positing and healing of the coracoid bone block, and no Hill–Sachs lesion, is a very rare but challenging situation.^25^ We report the case of a 21-year old female patient presenting with persistence of anterior shoulder instability after open Latarjet (performed after a failed arthroscopic Bankart) despite a well-positioned and fully healed coracoid bone block. Despite a reoperation to perform an open capsular shift (after screw removal), the patient could not use her arm because of painful dynamic and static anterior subluxation of the humeral head. The coracoid bone block was fully healed, no Hill–Sachs lesion, no fatty infiltration of the subscapularis, no axillary nerve palsy, no seizure nor any voluntary component that could explain the recurrence of anterior instability.

The question arises: what could we propose to this patient with recurrence of anterior shoulder instability despite a well-done Latarjet procedure and 2 previous failed soft tissue procedures (a failed Bankart and a failed capsular shift)? The surgical salvage options were limited: (1) the healing and the optimal positioning of the coracoid bone graft ruled out the use of another bone graft for glenoid reconstruction; (2) the absence of a Hill–Sachs lesion contraindicated a remplissage procedure, (3) the lack of an anterior labrum and the poor quality of the anterior capsule rendered new capsular and labrum reattachment unreliable, and (4) although early osteoarthritis was present, a reverse shoulder prosthesis^26^ or glenohumeral arthrodesis^8^^,^^28^ was deemed inappropriate for this young patient.

The fact that the recurrence of anterior shoulder instability was both dynamic (with recurrent subluxations) and static (with permanent decentering of the humeral head) led us hypothesizing that this could be related to elongation and plastic deformation of the subscapularis and the anterior capsule,^6^^,^^29^ and that performing a subscapularis and capsular shortening could restore shoulder stability.^11^ Based on this hypothesis, we performed a modified Putti–Platt procedure through a deltopectoral approach after an arthroscopic diagnosis that confirmed the impossibility to perform any labrum repair or a capsular shift. Postoperatively, our hypothesis was confirmed: at 2-year follow-up, the procedure proved successful, resulting in a stable, pain-free, and mobile shoulder. Postoperative CT scan images confirmed recentering of the humeral head in front of the glenoid surface (Figs. 6 and 7). This case demonstrates that a modified Putti–Platt procedure can serve as an effective salvage procedure for recurrence of anterior shoulder instability following multiple failed stabilization procedures, including a well-done Latarjet procedure with no glenoid or humeral bone loss.

The Putti–Platt procedure, described by Osmond–Clarke in 1948, is one of the oldest open ‘‘nonanatomic’’ technique proposed to stabilize the shoulder by shortening the subscapularis muscle and the anterior capsule.^22^ This procedure involves imbricating and shortening the subscapularis tendon and anterior capsule by medial advancement of the shoulder capsule and lateral advancement of the subscapularis, thereby enhancing the stability of the shoulder joint. This technique has been modified several times,^27^^,^^18^ and some series reported good outcomes^4^^,^^13^^,^^17^^,^^21^ but it has been practically abandoned due to some limitations of external rotation,^4^^,^^15^^,^^16^^,^^17^^,^^19^^,^^24^^,^^18^ high recurrence rate^10^^,^^13^^,^^21^ and development of osteoarthritis at long-term follow-up.^12^^,^^16^^,^^19^^,^^31^

Loss of range of motion, specifically in external rotation, and osteoarthritis have been labeled as the greatest disadvantages of the Putti–Platt procedure.^4^^,^^15^^,^^16^^,^^17^^,^^19^^,^^24^^,^^18^ At 2 years follow-up, our patient did not have significant limitation in external rotation (Fig. 4), and glenohumeral osteoarthritis did not increase, as it was already present (Fig. 5). It is our interpretation that the static humeral subluxation witnesses of the considerable plastic deformation of the subscapularis muscle and tendon in our patient, and that is why shortening of the elongated subscapularis was efficient to recenter the humeral head in front of the glenoid surface and did not limit range of motion or increase osteoarthritis.

It should be noticed that our technique is different than the one described with the original Putti–Platt procedure. With the classic Putti–Platt technique, the lateral capsular flap was separated from the subscapularis tendon and sutured to the anterior glenoid rim (labrum), which can significantly restrict external rotation.^11^^,^^29^ In our technique, we first sutured the medial flap to the lesser tuberosity to shorten the redundant subscapularis muscle and anterior capsule by 1.5 to 2 cm. This east–west shift of the capsule and tendon was then completed by suturing the lateral flap to the medial flap to create a double layer of tissue and prevent recurrence of anterior instability.

Despite its important role in shoulder instability, subscapularis muscle and tendon elongation has been neglected subjects in most stabilization procedures. At 2-year follow-up, the shoulder stability was maintained, motion restriction was marginal, and the patient was very satisfied to resume her daily activities and practice some sports. Radiographic evaluation at last follow-up showed that osteoarthritis had not progressed compared to before revision surgery and was not symptomatic.

This case demonstrates that (1) the role of the subscapularis in recurrence of anterior shoulder instability has been often overlooked: the elongation and plastic deformation of the subscapularis muscle and tendon may participate to recurrence or persistence of anterior instability; (2) the Latarjet procedure, despite the sling effect provided by the conjoint tendon, may be insufficient to reinforce the elongated anterior soft tissue structures and stabilize the shoulder in some hyperlax patients; and (3) The modified Putti–Platt procedure, by shortening the subscapularis muscle and tendon, may provide excellent shoulder stability without significant loss of range of motion, particularly in cases of recurrent shoulder instability where no humeral or glenoid bony deficiency can be identified.

The main limitation of our findings is that similar clinical scenarios are rare, and definitive conclusions cannot be drawn on a single case. Currently, there is no reliable diagnostic method to diagnose subscapularis insufficiency or elongation. However, we believe that the subscapularis muscle should be further investigated in the context of anterior shoulder instability to better understand its contribution to the underlying pathophysiology. Despite these limitations, this case highlights an important and underappreciated cause of recurrent anterior shoulder instability and demonstrates that, in the absence of bone loss, a modified Putti–Platt procedure can serve as a viable salvage option when traditional stabilization methods have failed.

## Conclusion

Our case report shows that, in the absence of glenoid or humeral bone loss, a modified Putti–Platt procedure can serve as an effective salvage procedure for recurrence of anterior shoulder instability following multiple failed stabilization procedures, including a well-done Latarjet procedure. This case demonstrates significant improvement in pain, function, and stability, suggesting that this procedure should be considered in similar clinical scenarios. This surgical technique should remain in the therapeutic arsenal of shoulder and orthopedic surgeons, as it can be very useful in specific clinical situations.

## Disclaimers:

Funding: No funding was disclosed by the authors.

Conflicts of interest: Pascal Boileau has received consulting fees from Smith & Nephew and royalties from Stryker that are not related to this study. The other authors, their immediate families, and any research foundations with which they are affiliated have not received any financial payments or other benefits from any commercial entity related to the subject of this article.

Patient consent: Obtained.
